# An Improved In-Motion Coarse Alignment Method for SINS/GPS Integration with Initial Velocity Error Suppression

**DOI:** 10.3390/s23073662

**Published:** 2023-03-31

**Authors:** Yukun Wang, Xiuli Ning, Xiang Xu

**Affiliations:** 1 School of Mechatronical Engineering, Beijing Institute of Technology, Beijing 100081, China; wyk521215@163.com; 2 China National Institute of Standardization, Beijing 100191, China; nxl_warm0908@163.com; 3 School of Automation, Nanjing University of Science and Technology, Nanjing 210094, China

**Keywords:** initial velocity errors, average operation for observation vectors, in-motion coarse alignment, SINS, GPS

## Abstract

The integrated system with the strapdown inertial navigation system (SINS) and the global positioning system (GPS) is the most popular navigation mode. It has been used in many navigation fields. Before the integrated system works properly, it must determine the initial attitude for SINS. In SINS/GPS-integrated systems, the navigational velocity can be used to carry out the initial alignment when the system is installed in the in-motion vehicle. However, the initial velocity errors are not considered in the current popular in-motion alignment methods for SINS/GPS integration. It is well-known that the initial velocity errors must exist when the initial velocity is obtained from the GPS outputs. In this paper, an improved method was proposed to solve this problem. By analyzing the original observation vectors in the in-motion coarse alignment method, an average operation was used to construct the intermediate vectors, and the new observation vector can be calculated by subtracting the intermediate vector from the original observation vector. Then, the initial velocity errors can be eliminated from the newly constructed observation vector. Thus, the interferences of the initial velocity errors for the initial alignment process can be suppressed. The simulation and field tests are designed to verify the performance of the proposed method. The tests results showed that the proposed method can obtain the higher accurate results than the current methods when the initial velocity is considered. Additionally, the results of the proposed method were similar to the current methods when the initial velocity errors were not considered. This shows that the initial velocity errors were eliminated effectively by the proposed method, and the alignment accuracy were not decreased.

## 1. Introduction

Navigation system is important for many applications, such as autonomous underwater vehicle (AUV), land vehicles, military systems and so on [1,2,3]. In many navigation systems, the strapdown inertial navigation system has the self-contained properties, and high sampling rates. Thus, SINS was found to be widely used for many applications [4,5]. However, the accumulated errors, which is caused by the measured errors of the inertial sensors, will be contained in the SINS outputs. Then, the positioning accuracy of SINS degrades. To address this problem, the SINS-based integrated navigation system was investigated [6,7]. Then, the accumulated errors can be corrected by the external measurements. In these integrated navigation systems, the SINS/GPS-integrated system is one of the most popular categories [8,9]. 

Before SINS/GPS works in full accuracy, the initial alignment process must be carried on for SINS. Many researchers were devoted to initial alignment research [10,11,12,13,14,15,16,17,18,19,20,21,22]. Firstly, the self-alignment methods in static or swaying base were investigated [10,11,12]. In [10], the attitude determination method with the Kalman filter was investigated. The convergence rate of the coarse alignment process was improved. In [11], the alignment errors were analyzed by an analytical calculation method when the SINS was on the stationary base. In [12], the parameter identification method was proposed for improving the vector observations. The random noises of the inertial sensors were weakened. These methods can be divided into the self-contained alignment process, because the alignment process can be finished only by the inertial sensors. The external equipment was not needed. Moreover, the self-alignment process can obtain the high accuracy during a short time, because the alignment process was not corrupted by the external interferences. However, the self-contained methods cannot be applicable to the in-motion situations, because the vehicle’s motion will interfere the extraction of the Earth gravitational apparent motion. Thus, the applied range of the self-contained alignment methods was limited. 

To extend the applied range, the in-motion alignment methods were devised [13,14,15,16,17]. In [13,14], the in-motion coarse alignment method for SINS/GPS integration was proposed, the detailed deduction of the observation vectors construction was described. With the SINS/GPS integration, the alignment results were high accuracy, which can be found in [13,14]. However, the outliers, which are contained in the GPS outputs, were ignored. Thus, the methods could not obtain the high accuracy when there were outliers in the GPS outputs. In [15], the position loci method was used to implement the in-motion alignment process. Based on the position loci, the outliers of GPS outputs were suppressed and the robustness of the in-motion alignment method was improved. These methods were based on the inertial frame theory. In [16,17], the in-motion alignment methods for SINS/DVL (Doppler Velocity Log) and SINS/OD (Odometer) systems were investigated, the alignment principles were also based on the inertial frame theory. Moreover, these in-motion alignment methods used the observation vectors, which were based on the chain rule of the direction cosine matrix (DCM). The alignment process was transformed to the attitude determination problem. However, it is noted that almost all aforementioned in-motion methods, which used the inertial frame theory, used the initial velocity of the vehicle to construct the observed vectors. Additionally, the initial velocity must be obtained from the external equipment since SINS worked in the initial alignment stage. However, the initial velocity errors were not concerned. Thus, there was a flaw in the proposed methods.

In this paper, we design an improved method for in-motion coarse alignment process, where the initial velocity errors are concerned. The intermediate observed vectors are introduced to construct the new observed vectors. Then, according to the vector subtraction operations, the initial velocity errors are eliminated from the new observed vectors. Based on the new observed vectors, the performance of the in-motion coarse alignment method is improved. In this paper, the proposed method is used for the SINS/GPS system. Additionally, we think it can be extended for other integration systems, such as SINS/DVL and SINS/OD integration. 

The rest of this paper is organized as follows. Section 2 explores the related works of the current in-motion coarse alignment for SINS/GPS integration. Then, the existing problems are analyzed. In Section 3, the construction for the new observation vectors is investigated. The simulation and field tests are designed to verify the performance of the proposed method in Section 4. Finally, the conclusions are drawn in Section 5.

## 2. Related Works and Problem Statements

In this section, the previous works will be reviewed and the observations vectors will be given. Moreover, the initial velocity errors will be introduced.

### 2.1. Related Works

The most popular methods for in-motion coarse alignment process are based on the inertial frame theory [13,14,15]. Thus, based on the inertial frame theory, the chain rule of the DCM can be given by:

(1) $$C_{b}^{n}(t)=C_{b(t)}^{n(t)}=C_{n(0)}^{n(t)}C_{b(0)}^{n(0)}C_{b(t)}^{b(0)}=C_{n(0)}^{n(t)}C_{b}^{n}(0)C_{b(t)}^{b(0)}$$

Additionally, the attitude rates of $C_{b(t)}^{b(0)}$ and $C_{n(0)}^{n(t)}$ are given by:

(2) $$\left\{\begin{array}{c} {\dot{C}}_{b(t)}^{b(0)}=C_{b(t)}^{b(0)}\left[\omega_{ib}^{b}\times \right]\\ {\dot{C}}_{n(t)}^{n(0)}=C_{n(t)}^{n(0)}\left[\omega_{in}^{n}\times \right] \end{array}\right.$$

where *b* denotes the body frame (*b*-frame), it is the right-forward-up (RFU) orthogonal frame in this paper; *n* denotes the navigation frame (*n*-frame), it is the east-north-up (ENU) orthogonal frame in this paper; *b*(0) and *n*(0) denote the *b*-frame and *n*-frame at the beginning of the alignment process, they are the non-rotating frame during the whole alignment procedure. In the alignment process, the angular velocity $\omega_{ib}^{b}$ can be obtained instead by the gyroscopes’ outputs. Additionally, the $\omega_{in}^{n}$ can be calculated by the velocity and position measurements of the GPS.

According to (1) and (2), only the DCM $C_{b}^{n}(0)$ is unknown. If $C_{b}^{n}(0)$ can be determined, the initial alignment can be finished. Referring to [13], the initial alignment process was transformed as the attitude determination problem with the observation vectors:

(3) $$C_{b}^{n}(0)\alpha_{v}=\beta_{v}$$

where

(4) $$\left\{\begin{array}{c} \alpha_{v}=\int_{0}^{t}C_{b(t)}^{b(0)}f^{b}dt\\ \beta_{v}=C_{n(t)}^{n(0)}v^{n}-v^{n}(0)+\int_{0}^{t}C_{n(t)}^{n(0)}\omega_{ie}^{n}\times v^{n}dt-\int_{0}^{t}C_{n(t)}^{n(0)}g^{n}dt \end{array}\right.$$

where $f^{b}$ denotes the specific force, it can be measured by the accelerometers; $\omega_{ie}^{n}$ denotes the Earth rate in *n*-frame; $g^{n}$ denotes the gravity in *n*-frame. It is noted that the reference vector $\alpha_{v}$ can be calculated by the outputs of accelerometers and gyroscopes in the in-motion coarse alignment process. Additionally, the observed vector $\beta_{v}$ can be calculated by the outputs of the GPS. 

Using the observation vectors, the ***K*** matrix can be constructed by:

(5) $$K=\sum {\left(\left[\beta_{v}⊗\right]-\left[\alpha_{v}⊙\right]\right)}^{T}\left(\left[\beta_{v}⊗\right]-\left[\alpha_{v}⊙\right]\right)$$

where

(6) $$\left\{\begin{array}{c} \left[\beta_{v}⊗\right]=\left[\begin{array}{cc} 0 & -\beta_{v}^{T}\\ \beta_{v} & \left[\beta_{v}\times \right] \end{array}\right]\\ \left[\alpha_{v}⊙\right]=\left[\begin{array}{cc} 0 & -\alpha_{v}^{T}\\ \alpha_{v} & -\left[\alpha_{v}\times \right] \end{array}\right] \end{array}\right.$$

According to Equations (5) and (6), the attitude quaternion can be extracted from the matrix ***K*** [15]. Thus, the DCM $C_{b}^{n}(0)$ can be obtained from the attitude quaternion. Then, the initial alignment process can be finished.

### 2.2. Problem Statement

Based on the aforementioned related works, which are shown in [13,14,15], the in-motion coarse alignment methods with SINS/GPS integration are implemented. Based on these methods, many researchers were devoted to extend the in-motion coarse alignment method to many other fields, such as underwater navigation vehicle [16,18], land vehicle and so on [19]. Additionally, the robust methods were proposed to address the outliers from the external auxiliary equipment, such as GPS, DVL and odometers [20,21,22]. However, from the observed vector $\beta_{v}$, it can be found that there exists an initial velocity $v^{n}(0)$ in the observation vector. When SINS is under in-motion coarse alignment process, the initial velocity can be obtained from the GPS outputs. Due to the specifications’ performance of the GPS, this initial velocity must be corrupted by the noises even outliers. If the initial velocity contains the errors, the alignment accuracy, which is based on the accuracy of the observation vector, will degrade. Moreover, in contrast to the real-time measurement errors of GPS, the initial velocity errors are the constant value during the whole alignment process. It is hard to use the outlier’s detection method to suppress them. Therefore, how to suppress the initial velocity errors is worth focusing on. We will give the simple effective method to solve this problem. The detailed method can be found in the next section.

## 3. The New Observation Vectors

According to the GPS measurements, the initial velocity from the GPS outputs can be modelled as:

(7) $${\tilde{v}}^{n}(0)=v^{n}(0)+\delta v_{0}^{n}$$

where ${\tilde{v}}^{n}(0)$ denotes the measured initial velocity from the GPS; $v^{n}(0)$ denotes the truth initial velocity; $\delta v_{0}^{n}$ denotes the initial velocity error. Substituting (7) into (4), we can obtain the calculated observed vector as:

(8) $$\begin{array}{ll} {\tilde{\beta }}_{v} & =C_{n(t)}^{n(0)}{\tilde{v}}^{n}-{\tilde{v}}^{n}(0)+\int_{0}^{t}C_{n(t)}^{n(0)}\omega_{ie}^{n}\times v^{n}dt-\int_{0}^{t}C_{n(t)}^{n(0)}g^{n}dt\\ & =C_{n(t)}^{n(0)}{\tilde{v}}^{n}-v^{n}(0)-\delta v_{0}^{n}+\int_{0}^{t}C_{n(t)}^{n(0)}\omega_{ie}^{n}\times v^{n}dt-\int_{0}^{t}C_{n(t)}^{n(0)}g^{n}dt\\ & ={\overset{⌣}{\beta }}_{v}-\delta v_{0}^{n} \end{array}$$

where ${\tilde{v}}^{n}$ denotes the real-time measured velocity from GPS; and ${\overset{⌣}{\beta }}_{v}=\beta_{v}+C_{n(t)}^{n(0)}\delta v_{}^{n}$. It is noted that ${\tilde{v}}^{n}$ also contain the noises, but it is not a constant value during the whole alignment process, and it can be suppressed by the robust methods [22]. In this study, we only focused on the initial velocity errors.

From [13,22], the following equation was obtained:

(9) $${\overset{⌣}{\beta }}_{v}=C_{b}^{n}(0){\tilde{\alpha }}_{v}$$

where

(10) $${\tilde{\alpha }}_{v}=\int_{0}^{t}{\tilde{C}}_{b(t)}^{b(0)}{\tilde{f}}^{b}dt$$

Additionally, ${\tilde{C}}_{b(t)}^{b(0)}$ and ${\tilde{f}}^{b}$ are obtained from the gyroscopes and accelerometers. It is noted that the upper tilde means there are errors in ${\tilde{C}}_{b(t)}^{b(0)}$ and ${\tilde{f}}^{b}$ with the inertial sensors.

From (8), using the discrete form of the observation vectors, the averaged observed vectors can be calculated by:

(11) $$\begin{array}{ll} {\bar{\beta }}_{v,M} & =\frac{1}{M}\sum_{k=1}^{M}{\tilde{\beta }}_{v,k}\\ & =\frac{1}{M}\sum_{k=1}^{M}\left({\overset{⌣}{\beta }}_{v,k}-\delta v_{0}^{n}\right)\\ & =\frac{1}{M}\sum_{k=1}^{M}{\overset{⌣}{\beta }}_{v,k}-\delta v_{0}^{n} \end{array}$$

where the subscript *k* denotes the vectors at time instant *k*; The *M* denotes the current time instant *M*.

Using (7), the new observed vector can be calculated by the vector subtraction:

(12) $$\begin{array}{ll} {\hat{\beta }}_{v,M} & ={\tilde{\beta }}_{v,M}-{\bar{\beta }}_{v,M}\\ & ={\tilde{\beta }}_{v,M}-\frac{1}{M}\sum_{k=1}^{M}{\tilde{\beta }}_{v,k}\\ & ={\overset{⌣}{\beta }}_{v,M}-\delta v_{0}^{n}-\left(\frac{1}{M}\sum_{k=1}^{M}{\overset{⌣}{\beta }}_{v,k}-\delta v_{0}^{n}\right)\\ & ={\overset{⌣}{\beta }}_{v,M}-\frac{1}{M}\sum_{k=1}^{M}{\overset{⌣}{\beta }}_{v,k} \end{array}$$

Based on (9), it was:

(13) $${\hat{\beta }}_{v,M}=C_{b}^{n}(0){\hat{\alpha }}_{v,M}$$

where

(14) $${\hat{\alpha }}_{v,M}={\tilde{\alpha }}_{v,M}-\frac{1}{M}\sum_{k=1}^{M}{\tilde{\alpha }}_{v,k}$$

From the above deduction, it can be found that the initial velocity errors $\delta v_{0}^{n}$ are eliminated from the new observed vectors ${\hat{\beta }}_{v,M}$. Thus, using the new observed vector ${\hat{\beta }}_{v,M}$, the alignment results will be more accurate than the previous method, which the initial velocity errors $\delta v_{0}^{n}$ are not eliminated. Using the new observation vectors ${\hat{\beta }}_{v,M}$ and ${\hat{\alpha }}_{v,M}$, the initial attitude $C_{b}^{n}(0)$ can be determined. Then, the initial alignment can be finished with (1). 

The algorithm flowchart of the proposed method is summarized in Algorithm 1. From Algorithm 1, it can be divided into three parts. Firstly, the initial parameters are needed to set. The initial parameters contain ***K*** matrix, DCM, and vector observations. Secondly, the outputs of the inertial sensors and the GPS are obtained. Using the outputs information, the alignment process can be carried out. Thirdly, the alignment process is carried out. Additionally, the alignment results are obtained at real-time.
**Algorithm 1.** Initial alignment method for SINS/GPS integration**Initialization:** $K_{0}=0,C_{b}^{b0}(0)=C_{n}^{n0}(0)=I_{3},{\tilde{\alpha }}_{v,0}={\tilde{\beta }}_{v,0}=0$,**Inputs:** ${\left\{f^{b}\right\}}_{k=1}^{N},{\left\{\omega_{ib}^{b}\right\}}_{k=1}^{N},{\left\{{\tilde{v}}^{n}\right\}}_{k=1}^{N}$**for:** *k* = 1, 2, 3… **do**      Calculate $C_{n(k)}^{n0}$ and $C_{b(k)}^{b0}$ using (2)      **if** GPS outputs are available             Calculate the observation vectors ${\tilde{\alpha }}_{v,k}$ and ${\tilde{\beta }}_{v,k}$ by (4)             Calculate the averaged observation vector according to (7)             ${\bar{\beta }}_{v,M}=\frac{1}{M}\sum_{k=1}^{M}{\tilde{\beta }}_{v,k}$             Calculate the new observed vector by (8)             ${\hat{\beta }}_{v,M}={\tilde{\beta }}_{v,M}-{\bar{\beta }}_{v,M}$             Calculate the averaged reference vector according to (10)             ${\hat{\alpha }}_{v,M}={\tilde{\alpha }}_{v,M}-\frac{1}{M}\sum_{k=1}^{M}{\tilde{\alpha }}_{v,k}$             Construct the matrix             $K_{M}=K_{M-1}+{\left(\left[{\hat{\beta }}_{v,M}⊗\right]-[{\hat{\alpha }}_{v,M}⊙]\right)}^{T}\left(\left[{\hat{\beta }}_{v,M}⊗\right]-[{\hat{\alpha }}_{v,M}⊙]\right)$             Extract $q_{n0}^{b0}$ from ***K*_*M*_**, and transform $q_{b0}^{n0}$ to $C_{n0}^{b0}$      **end if**      Calculate the current attitude according to      $C_{b}^{n}(k)=C_{n0}^{n(k)}C_{b0}^{n0}C_{b(k)}^{b0}$     *k* = *k* + 1**end for**

## 4. Simulation and Field Test Results

To verify the performance of the proposed method, the simulation and field tests were designed in this section. The alignment results are shown in the next two subsections. The current popular methods, which were proposed in [13,15], were designed as the comparative methods and four different initial velocity errors are considered.

Scheme 1: The current popular method [13] with the initial velocity errors [5 5 5] m/s.

Scheme 2: The current popular method [13] with the initial velocity errors [1 1 1] m/s.

Scheme 3: The current popular method [13] with the initial velocity errors [0.1 0.1 0.1] m/s.

Scheme 4: The current popular method [15] with the initial velocity errors [5 5 5] m/s.

Scheme 5: The current popular method [13] without the initial velocity errors.

Scheme 6: the proposed method with the initial velocity error [5 5 5] m/s.

### 4.1. Simulation Test

**Figure 1:**
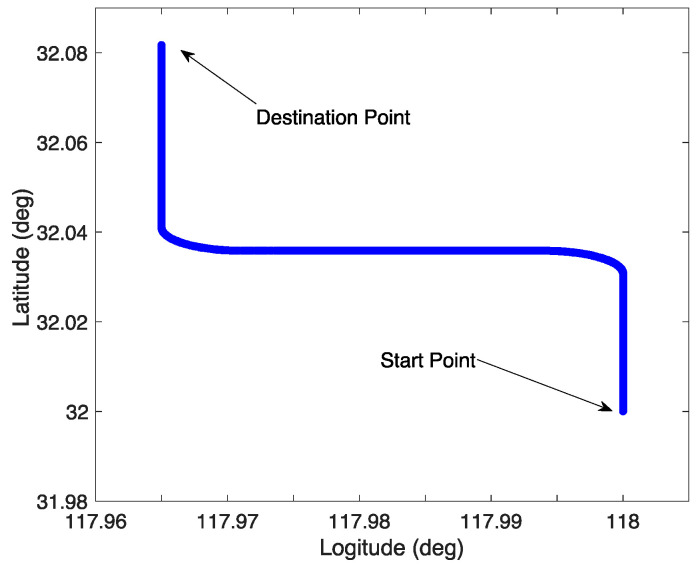
The in-motion trajectory of the vehicle.

**Figure 2:**
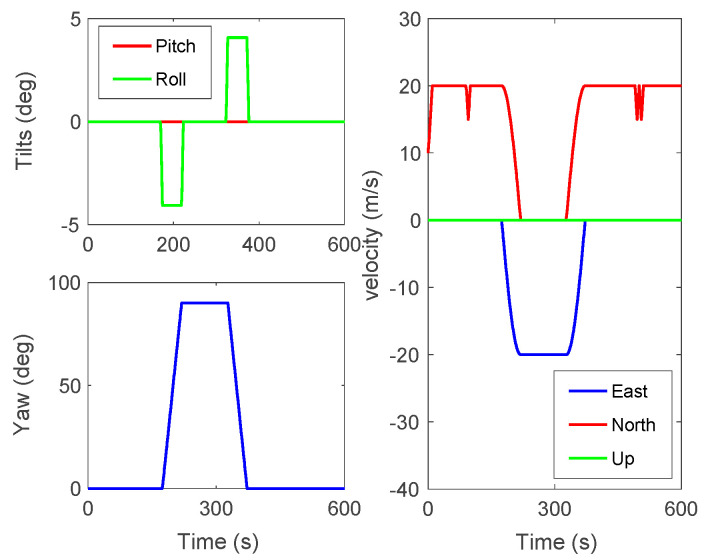
The in-motion status of the vehicle.

In the simulation tests, the Zigzag trajectory was designed for verification. The trajectory and the movement states of the vehicle are shown in Figure 1 and Figure 2. The initial position of the vehicle was set as L = 32° N, λ = 118° E, where L and λ denote the latitude and longitude, respectively.

The bias and the angular rate random walk of the gyroscopes were set as 0.01°/h and 0.005°/√h. The bias and the velocity random walk of the accelerometers were set as 100 μg and 50 μg/√Hz. The sampling rate of inertial sensors was set as 200 Hz. The velocity noise of GPS was set as 0.1 m/s and the position noises of GPS was set as 10 m. The noises of GPS were correspondent to Gaussian distribution. The sampling rate of the GPS was set as 1 Hz.

**Figure 3:**
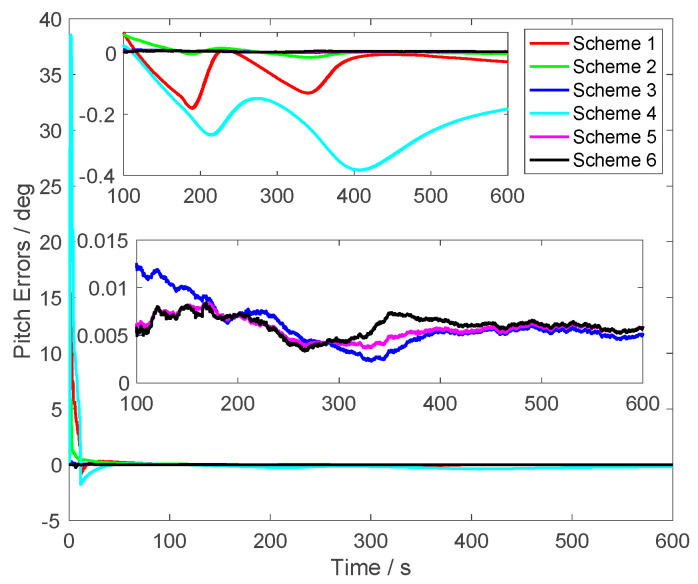
The pitch errors.

**Figure 4:**
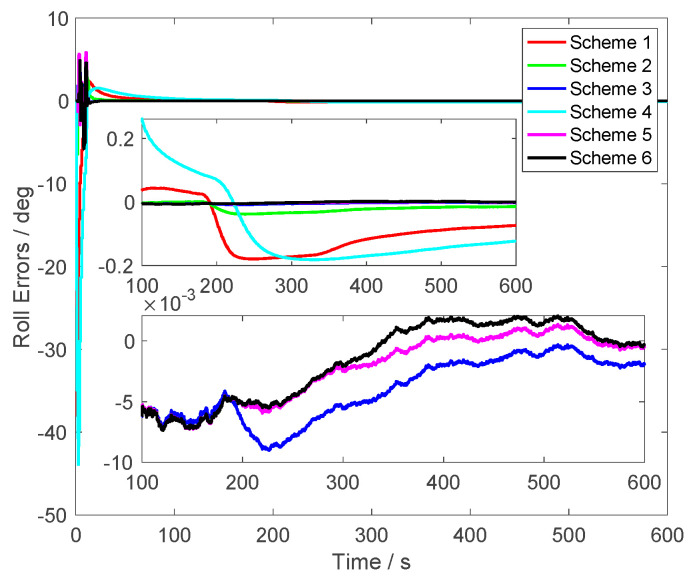
The roll errors.

**Figure 5:**
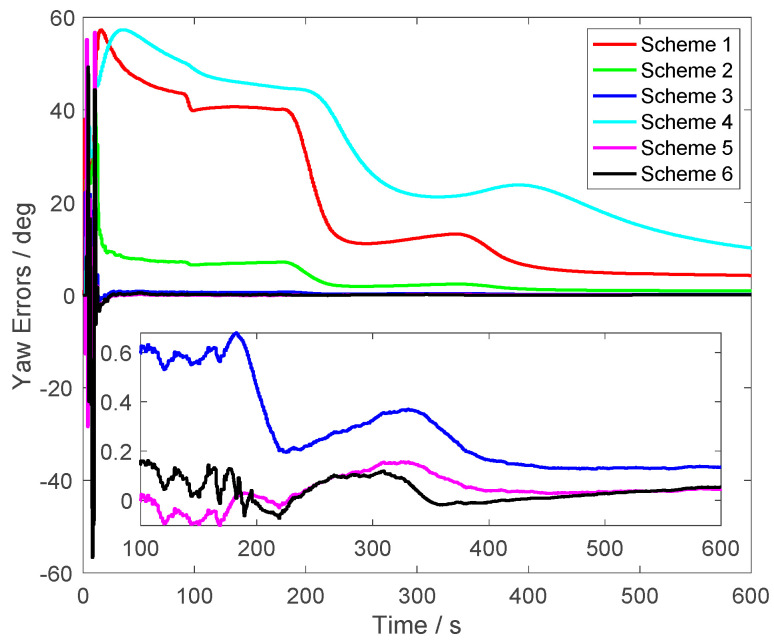
The yaw errors.

The alignment results are shown in Figure 3, Figure 4 and Figure 5. In Figure 3, the pitch errors are presented; it was found that the alignment errors of Schemes 1 and 4 were worse than other schemes. This is because the initial velocity errors in Schemes 1 and 4 were set as [5 5 5] m/s. The alignment errors of Scheme 2 were less than the same one of Schemes 1 and 4. This is because the initial velocity errors of Scheme 2 were set as [1 1 1] m/s. However, with the same initial velocity errors [5 5 5] m/s, the proposed method, which is Scheme 6, can obtain the high accuracy alignment results of the pitch. From Figure 3, it was found that the alignment errors of the proposed method were around 0.005°. The alignment results were close to Scheme 5, in which the initial velocity errors were not set. Additionally, the alignment results of Scheme 3 were also close to Scheme 5 because the initial velocity was set as [0.1 0.1 0.1] m/s, which is similar to the noises of the GPS outputs. These results showed that the different initial velocity errors will produce different alignment results. The larger initial velocity errors were contained in the GPS outputs and the large alignment errors will be contained in the alignment results of the traditional methods.

In Figure 4, similar results were found, the roll errors of Scheme 5 were less than 0.005° after 200 s. The roll errors of Scheme 1 and Scheme 4 were larger than 0.1°. Although the alignment errors of Scheme 2 were less than the same one of Scheme 1 and Scheme 4, they were still larger than the proposed method. When alignment process lasts 200 s, compared with the alignment errors of Scheme 3, it was found that the roll errors of Scheme 6, which was the proposed method, were closer to the alignment results of Scheme 5. This conclusion shows that the proposed method could suppress the initial velocity errors effectively. It is noted that the roll errors of Scheme 6 had a similar convergence rate with the errors of Scheme 5. This conclusion showed that the vector subtraction operations did not weaken the characteristics of the vector observations. 

In Figure 5, the yaw errors are shown. From Schemes 1, 2 and 4, it was found that the yaw accuracy was degraded by the initial velocity errors. In Scheme 1, the errors were larger than 5° when the alignment process lasted for 300 s. In Scheme 2, the errors were smaller than the errors of Scheme 1. This is because the initial velocity errors were small in Scheme 2. However, the errors were still larger than the errors of the proposed method. Although the alignment errors of Scheme 3 were less than 0.5°, they were still larger than the alignment errors of Scheme 5, which were not corrupted by the initial velocity errors. At the end of alignment process, the yaw errors were around 0.1° of Schemes 5 and 6. From the enlarged figure in Figure 5, it was found that the curve of Scheme 6, which was the proposed method, was consistent with the results of Scheme 5. This conclusion showed that the initial velocity errors were eliminated by the proposed method effectively. Moreover, the convergence rate of Scheme 6 was not degraded. 

From Figure 3, Figure 4 and Figure 5, it was found that the curves of the alignment errors of Schemes 5 and 6 were coincidental. These results show that the proposed method can eliminate the initial velocity error effectively.

### 4.2. Field Test

**Figure 6:**
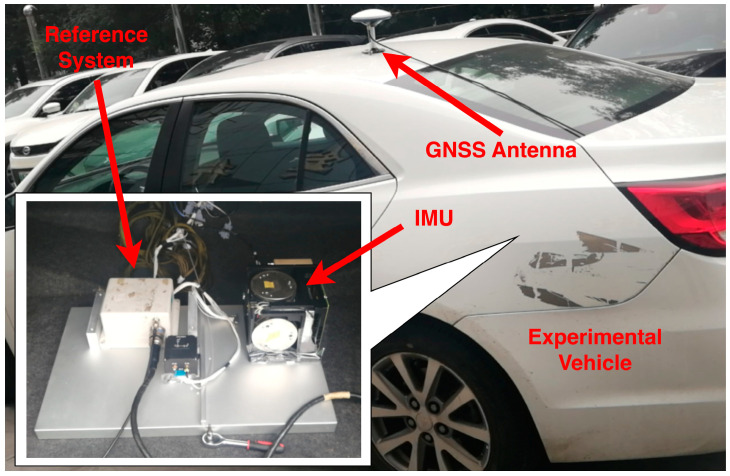
The experimental vehicle and equipment.

To show the performance of the proposed method in practical systems, the field test was designed. The experimental vehicle and equipment are shown in Figure 6. The navigational computer was produced by our team. It was combined with a PC104 board. The CPU (central processing unit) can operate up to 500 MHz. The GPS receiver was produced by the NovAtel, the BESTVEL and BESTPOS logs were used to output the velocity and position of the vehicle, the sampling rate of the GPS was set as 1 Hz. The reference system, which is named CPT7, was produced by NovAtel. The accuracies of the pitch, roll and yaw of CPT7 were 0.01°, 0.01° and 0.1°, respectively. The specifications of inertial measurement unit (IMU), which were combined with triaxial accelerometers and gyroscopes, are shown in Table 1.

**Figure 7:**
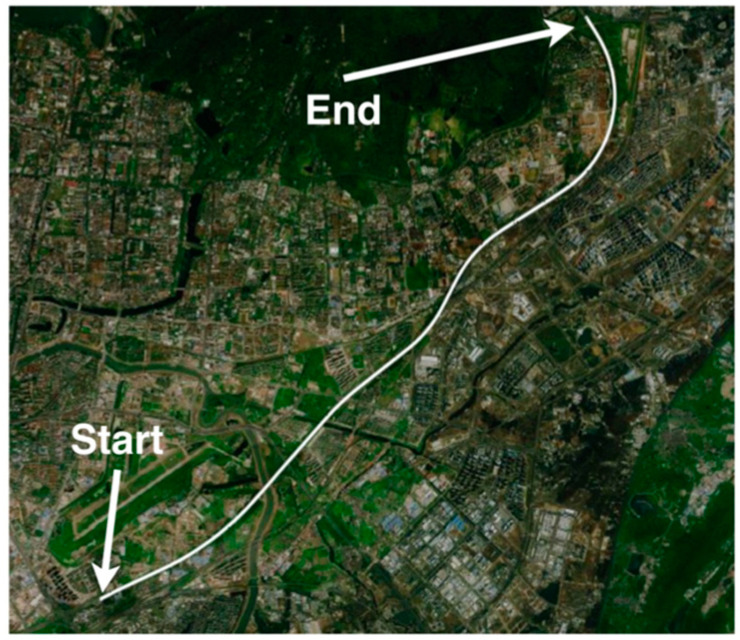
The in-motion trajectory of the vehicle.

**Figure 8:**
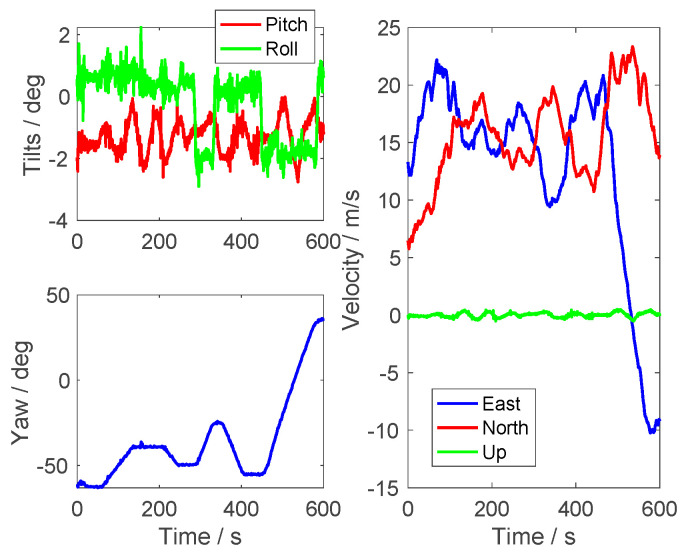
The in-motion status of the vehicle.

**Figure 9:**
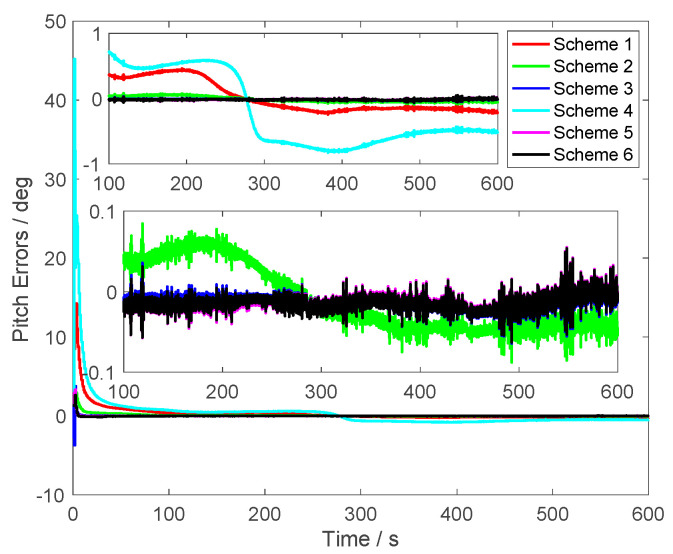
The pitch errors.

**Figure 10:**
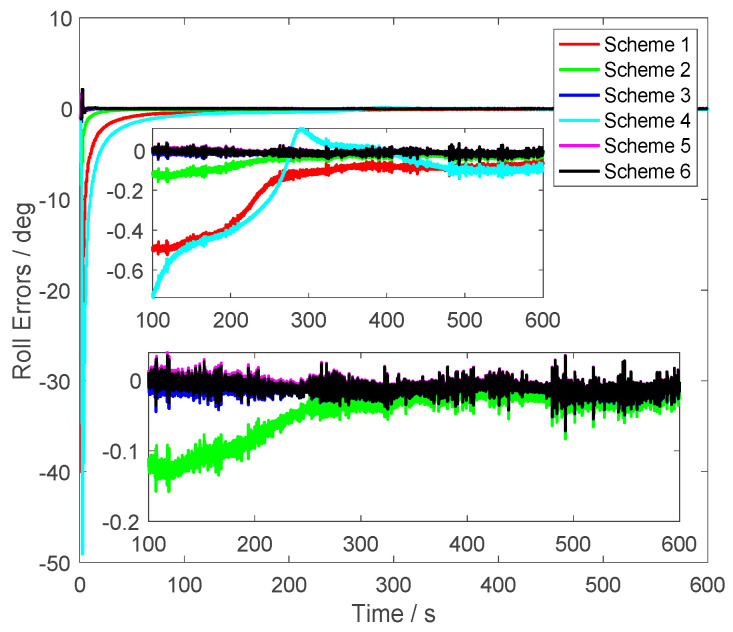
The roll errors.

**Figure 11:**
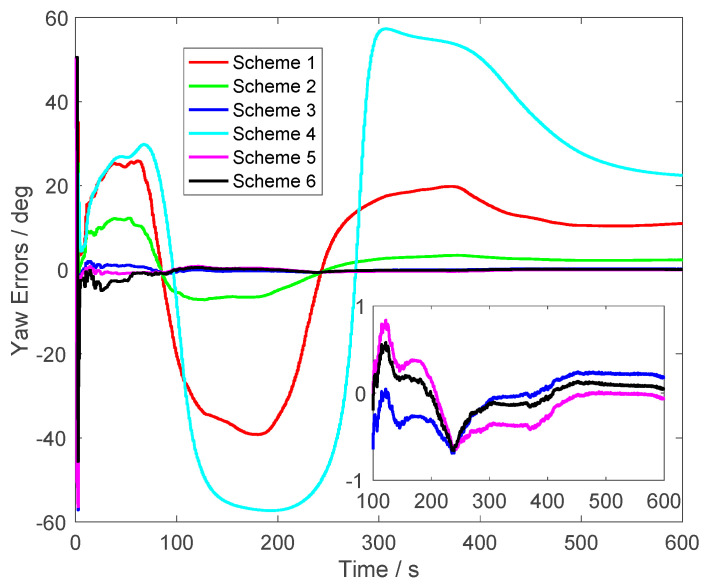
The roll errors.

It was found that the specification of the inertial sensors was not a determined value; this is because the errors of the inertial sensors were time-varying. The moving trajectory and the states of the vehicle are shown in Figure 7 and Figure 8. The averaged moving velocity was around 20 m/s. Additionally, the alignment process was carried out when the vehicle was moving at any time. The alignment results are shown in Figure 9, Figure 10 and Figure 11.

In Figure 9, the pitch errors are shown. Due to the big initial velocity errors, it was found that the alignment errors of Schemes 1 and 4 were larger than 0.1°. The results were unsatisfactory. The pitch errors of Scheme 2 were around 0.05° when the alignment time lasted 150 s. However, it was also larger than the proposed method, which was shown as Scheme 6. The errors of Schemes 3, 5 and 6 were closer to each other. In Scheme 3, the initial velocity errors were set as [0.1 0.1 0.1] m/s. Additionally, there were no initial velocity errors in Scheme 5. However, the proposed method, which was Scheme 6, contained the initial velocity errors, which were [5 5 5] m/s. These results showed that the initial velocity errors were suppressed by the proposed method. The alignment errors of pitch of Scheme 6 were less than 0.01°.

In Figure 10, the roll errors are shown. The errors of Schemes 1 and 4 were also fluctuant. This was because the initial velocity errors were large, and there was no effective method to suppress them. The errors of Schemes 1 and 4 were around 0.1° when the alignment process lasted 300 s. The errors of Scheme 2 were fewer than the errors of Schemes 1 and 4. This was because the initial velocity errors were small. However, they were still worse than the errors of Scheme 6. The errors of Schemes 3, 5 and 6 were similar, i.e., they were less than 0.01°. In Scheme 3, the initial velocity errors were 0.1 m/s. Additionally, in Scheme 5, the initial velocity errors were 0 m/s. However, in Scheme 6, which was the proposed method, the initial velocity errors were 5 m/s. Compared with the results of Schemes 3 and 5, it was found that the large initial velocity errors in Scheme 6 were suppressed.

In Figure 11, the yaw errors are shown. The errors of Schemes 1 and 4 were large and were caused by the relatively large initial velocity errors. Moreover, the alignment errors of Scheme 2 were larger than 2° when the alignment time lasted 300 s. The other three methods, which were Schemes 3, 5 and 6, had the similar alignment results. However, it is noted that the initial velocity errors in Schemes 3 and 5 were small. Moreover, the alignment errors of Scheme 6 were closer to Scheme 5 than Scheme 3. It was shown that although the yaw errors of Scheme 3 could converge towards less than 0.5°, they were also affected by the initial velocity errors. Thus, the alignment errors were a little bigger than the proposed method. After 300 s, the yaw errors of Scheme 6 were less than 0.2°, and 5 m/s initial velocity errors were contained in Scheme 6. The field test also verified the performance of the proposed method.

## 5. Conclusions

In this paper, an improved method for in-motion coarse alignment process of SINS/GPS integration was proposed. The initial velocity errors, which were contained in the observed vectors, were considered. If the initial velocity errors were contained in the observed vectors, the alignment error would have been large. To address this problem, the average operation was used to construct the intermediate observed vectors. Then, the new observed vectors were constructed by the intermediate observed vectors with the vector subtraction operations. In the new observed vectors, the initial velocity errors were eliminated effectively. Thus, the alignment accuracy was improved. Moreover, the characteristics of the vector observations were reserved. The simulation and field tests were designed to verify the performance of the proposed method. The results showed that the proposed method could suppress the influences of initial velocity errors on the initial alignment procedure. The proposed method can also be used in other in-motion coarse alignment processes.

## Figures and Tables

**Table 1:** The specification of the inertial sensors.

Error Items	Gyroscopes (x-, y-, z-Axes)	Accelerometers (x-, y-, z-Axes)
Bias	≤(0.05, 0.05, 0.05)°/h	≤(1000, 1000, 1000) μg
Random Walk Coefficients	≤(0.005, 0.005, 0.005)°/√h	≤(50, 50, 50) μg/√Hz

## Data Availability

Not applicable.

## References

[1] Groves P.D. (2013). Principles of GNSS, Inertial, and Multi-Sensor Integrated Navigation Systems.

[2] Wu Y., Pan X. (2013). Velocity/position integration formula part II: Application to strapdown inertial navigation computation. IEEE Trans. Aerosp. Electron. Syst..

[3] Gao W., Ben Y., Zhang X., Li Q., Yu F. (2011). Rapid fine strapdown INS alignment method under marine mooring condition. IEEE Trans. Aerosp. Electron. Syst..

[4] Qin F., He H., Xu J. (2018). Phase Modulation-Based SINS Damping Method for Autonomous Vehicles. IEEE Sens. J..

[5] Yao Y., Xu X., Li Y., Liu Y., Sun J., Tong J. (2016). Transverse Navigation under the Ellipsoidal Earth Model and its Performance in both Polar and Non-polar areas. J. Navig..

[6] Wang B., Yu L., Deng Z., Fu M. (2016). A particle filter-based matching algorithm with gravity sample vector for underwater gravity aided navigation. IEEE/ASME Trans. Mechatron..

[7] Shen C., Bai Z., Cao H., Xu K., Wang C., Zhang H., Wang D., Tang J., Liu J. (2016). Optical flow sensor/INS/magnetometer integrated navigation system for MAV in GPS-denied environment. J. Sens..

[8] Hong S., Lee M.H., Kwon S.H., Chun H.H. (2004). A car test for the estimation of GPS/INS alignment errors. IEEE Trans. Intell. Transp. Syst..

[9] Hong S., Lee M.H., Chun H., Kwon S., Speyer J.L. (2006). Experimental study on the estimation of lever arm in GPS/INS. IEEE Trans. Veh. Technol..

[10] Xu X., Xu X., Zhang T., Li Y., Tong J. (2017). A Kalman Filter for SINS Self-Alignment Based on Vector Observation. Sensors.

[11] Silva F.O., Hemerly E.M., Waldemar Filho C.L. (2016). Error analysis of analytical coarse alignment formulations for stationary SINS. IEEE Trans. Aerosp. Electron. Syst..

[12] Xu X., Xu X., Zhang T., Li Y., Zhou F. (2016). Improved Kalman filter for SINS coarse alignment based on parameter identification. Zhongguo Guanxing Jishu Xuebao/J. Chin. Inert. Technol..

[13] Wu Y., Pan X. (2013). Velocity/Position integration formula part I: Application to in-flight coarse alignment. IEEE Trans. Aerosp. Electron. Syst..

[14] Zhang T., Zhu Y., Xu X., Wang J., Li Y. (2018). In-motion coarse alignment based on the vector observation for SINS. IEEE Trans. Instrum. Meas..

[15] Xu X., Xu D., Zhang T., Zhao H. (2019). In-Motion Coarse Alignment Method for SINS/GPS Using Position Loci. IEEE Sens. J..

[16] Xu X., Guo Z., Yao Y., Zhang T. (2020). Robust Initial Alignment for SINS/DVL Based on Reconstructed Observation Vectors. IEEE/ASME Trans. Mechatron..

[17] Huang Y., Zhang Y., Wang X. (2017). Kalman-Filtering-Based In-Motion Coarse Alignment for Odometer-Aided SINS. IEEE Trans. Instrum. Meas..

[18] Xu X., Sun Y., Gui J., Yao Y., Zhang T. (2020). A Fast Robust In-Motion Alignment Method for SINS With DVL Aided. IEEE Trans. Veh. Technol..

[19] Li J., Gao W., Zhang Y. (2018). Gravitational apparent motion-based SINS self-alignment method for underwater vehicles. IEEE Trans. Veh. Technol..

[20] Xu X., Gui J., Sun Y., Yao Y., Zhang T. (2020). A robust in-motion alignment method with inertial sensors and Doppler velocity log. IEEE Trans. Instrum. Meas..

[21] Sun Y., Yang G., Cai Q., Wen Z. (2020). A robust in-motion attitude alignment method for odometer-aided strapdown inertial navigation system. Rev. Sci. Instrum..

[22] Xu X., Sun Y., Yao Y., Zhang T. (2021). A Robust In-Motion Optimization-Based Alignment for SINS/GPS Integration. IEEE Trans. Intell. Transp. Syst..

